# Evaluation of Disk Halo Size after Implantation of a Collamer Lens with a Central Hole (ICL V4c)

**DOI:** 10.1155/2019/7174913

**Published:** 2019-08-14

**Authors:** Xun Chen, Tian Han, Feng Zhao, Huamao Miao, Xiaoying Wang, Xingtao Zhou

**Affiliations:** ^1^Department of Ophthalmology, Eye and ENT Hospital of Fudan University, Shanghai, China; ^2^NHC Key Laboratory of Myopia (Fudan University), Shanghai, China; ^3^Research Center of Ophthalmology and Optometry Shanghai, Shanghai, China; ^4^The Department of Ophthalmology, Shuguang Hospital of Shanghai University of Traditional Chinese Medicine, Shanghai, China

## Abstract

**Purpose:**

To investigate disk halo size changes produced by a glare source after surgical insertion of an implantable collamer lens with a central hole (ICL V4c) for myopia correction.

**Methods:**

In this prospective study, disk halo size and pupillary light response with a vision monitor were measured preoperatively and at 1 week, 1 month, and 3 months postoperatively. Pupillary light response parameters included contraction amplitude, latency, duration, and velocity; dilation latency, duration, and velocity; and initial, maximum, minimum, and average pupil diameters.

**Results:**

Forty-two right eyes of 42 patients were enrolled. Postoperative uncorrected distance visual acuity was better than or equal to 20/20 in all eyes. Compared to preoperative values, disk halo size showed no significant difference at 1 week postoperatively (*P* > 0.05) and then decreased significantly at 1 and 3 months postoperatively (both *P* < 0.001). Contraction amplitude and velocity, as well as dilation velocity, decreased significantly at all postoperative time points (all *P* < 0.001). Disk halo size at 3 months postoperatively was significantly correlated with initial (*r* = 0.446, *P*=0.003), maximum (*r* = 0.483, *P*=0.001), minimum (*r* = 0.425, *P*=0.005), and average pupil diameters (*r* = 0.474, *P*=0.002).

**Conclusions:**

After ICL V4c implantation, disk halo size was reduced in the short term. Patients with smaller pupil sizes during pupillary response to light experienced smaller halos after ICL V4c implantation.

## 1. Introduction

The implantable collamer lens with a central hole (ICL V4c; STAAR Surgical Company, Monrovia, CA, USA) is a new posterior chamber intraocular lens (IOL) that is designed to allow natural flow of the aqueous humor from the posterior chamber to the anterior chamber through a 360 *μ*m central hole; notably, it does not require a preoperative peripheral iridotomy, which contrasts with the approach necessary for a conventional ICL V4. Although the ICL V4c has shown excellent clinical and refractive results when used for the treatment of myopia [1–5], some patients may continue to experience postoperative halos. In addition, with the refinement of refractive surgeries, surgeons have pursued both vision enhancement and maintenance of visual quality, including the prevention of halos. Halos develop when a strong light source is located in the visual field, such that forward-scattered light in the eye produces a veiling light over the retina. Halos may cause many complications in daily life and alter visual quality, especially for patients driving at night. Thus far, there have been few studies of halos induced by a glare source after ICL implantation.

A previous study showed that in myopic patients, the minimum pupil size achieved within the dynamic pupillary light response was correlated with disk halo size [6]. Considering that the dynamic pupillary light response changes after ICL implantation [7–9], there is a need to investigate the relationship between disk halo size and dynamic pupillary light response after ICL V4c implantation.

The present study was performed to investigate disk halo size changes using a visual monitor with good repeatability [10], as well as their relationships with dynamic pupillary light response after ICL V4c implantation.

## 2. Materials and Methods

This study was approved by the Ethics Committee of the Eye and ENT Hospital of Fudan University and complied with tenets of the Declaration of Helsinki. Informed written consent was obtained from all patients after the possible consequences of the study were explained.

### 2.1. Study Population

This prospective study enrolled consecutive patients who underwent routine preoperative ophthalmic examinations at the Refractive Surgery Centre in the Department of Ophthalmology of the Eye and ENT Hospital of Fudan University. The inclusion criteria were as follows: age between 20 and 35 years, stable refraction, anterior chamber depth ≥2.8 mm, and endothelial cell density ≥2,000 cells/mm^2^. Corrected distance visual acuity was better than or equal to 20/20 in all patients. Because age is associated with both the pupillary response to light and disk halo size [11], and patients between 20 and 50 years of age have similar disk halo sizes [10], the criteria were modified to include only patients aged 20–35 years. The exclusion criteria were as follows: a history of ocular diseases other than myopia and astigmatism (corneal or lens opacity, retinal detachment, glaucoma, macular degeneration, or neuro-ophthalmic disease); a history of ocular surgery, or trauma; and/or a history of systemic diseases.

### 2.2. Surgical Procedure

ICL V4c implantation procedures were performed by two experienced surgeons (X. Y. W. and X. T. Z.). As described in a previous study [12], the viscoelastic surgical agent was injected into the anterior chamber via a puncture site at the 6 o'clock position of the cornea; subsequently, an ICL V4c was implanted through a 3.0 mm temporal corneal incision using an injector cartridge and then placed in the posterior chamber. Next, the viscoelastic surgical agent was completely replaced by a balanced salt solution.

### 2.3. Measurements

Disk halo size and pupillary light response were evaluated by an experienced technician preoperatively and at 1 week, 1 month, and 3 months postoperatively with monocular dynamic pupillometry (MonCv3; Metrovision, Pérenchies, France). As described in previous studies [10, 13], all measurements were obtained between 9:00 AM and 12:00 AM after 5 min of darkness adaptation. A light source on the right side was used to test the right eye with a luminance of 5 cd/m^2^. In preoperative measurements, a lens was used to achieve full correction of refractive error. Three radial lines of 10 letters appeared from the periphery toward the light source on the screen, such that the 10 letters formed 10 rings at 30 min arc intervals (arcmin). The average distance to the letter nearest to the light source was measured for each line, and then the visual angle formed by the radius of the halo was calculated in arcmin. The pupillary contour was automatically traced by the pupillometer with an accuracy of ±0.1 mm. The software then performed an analysis of responses to successive visual stimuli with automated quantification of the following parameters: contraction amplitude, latency, duration, and velocity; dilation latency, duration, and velocity; and initial, maximum, minimum, and average pupil diameters. Each parameter was measured at least five times and the mean values were recorded.

### 2.4. Statistical Analysis

All statistical analyses were performed using the Statistical Package for Social Sciences (version 22; IBM Corp., Armonk, NY, USA). All data were tested for normality using the Kolmogorov–Smirnov test. A repeated-measures analysis of variance with least significant difference post hoc comparisons and the Friedman test were performed to evaluate temporal changes in disk halo size and pupillary response to light. Pearson's and Spearman's correlation analyses were applied to detect potential correlations between these variables. A *P* value < 0.05 was considered to be statistically significant.

## 3. Results

Forty-two consecutive patients (female-to-male ratio = 24 : 18) were enrolled; these patients had a mean age of 24.76 ± 5.16 years and mean spherical equivalent (SE) of −10.42 ± 2.54 diopters.  Table 1 shows the demographic and refractive data. All surgeries were uneventful, and no intraoperative or postoperative complications were observed. No patients failed to follow-up in this study. Postoperative uncorrected distance visual acuity was better than or equal to 20/20 in all patients.

**Figure 1:**
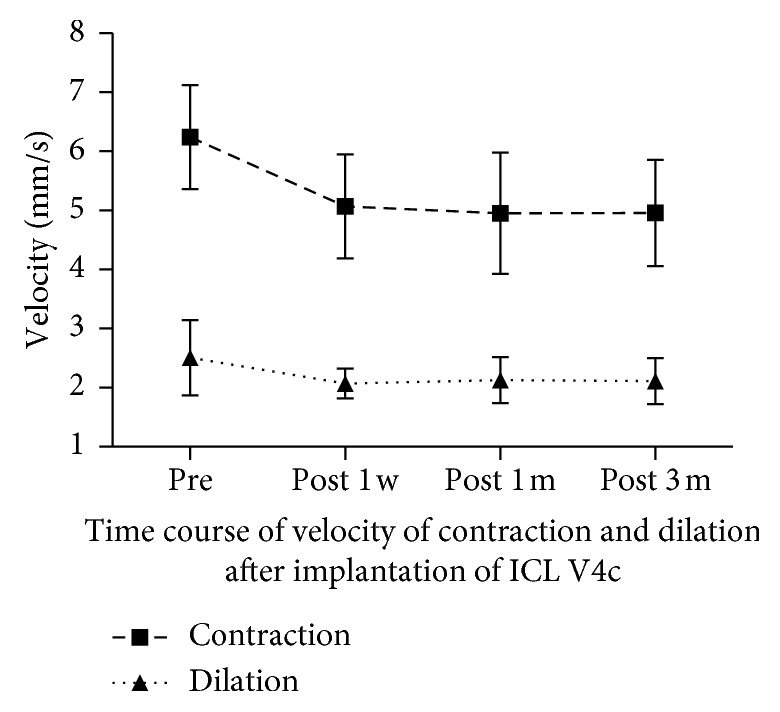
Changes in the velocities of contraction and dilation after ICL V4c implantation. Compared to preoperative values, the velocities of contraction and dilation are decreased significantly at 1 week, 1 month, and 3 months postoperatively (all *P* < 0.001).

Preoperative and postoperative disk halo size and pupillary response to light measurements are shown in Table 2. Compared to preoperative values, disk halo size decreased significantly at 1 and 3 months postoperatively (both *P* < 0.001), but showed no significant difference at 1 week postoperatively (*P* > 0.05). Contraction amplitude and velocity, as well as dilation velocity, decreased significantly at all postoperative time points, compared to preoperative values (all *P* < 0.001); there were no differences among postoperative time points (all *P* > 0.05) (Figure 1). No significant changes were observed in contraction latency, contraction duration, dilation latency, or dilation duration between any time points (all *P* > 0.05).

**Figure 2:**
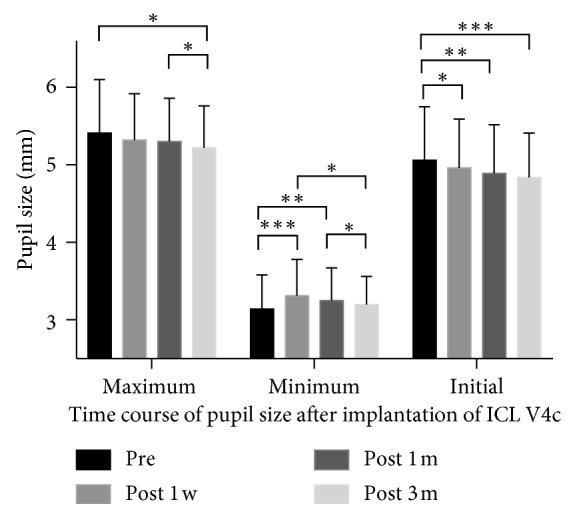
Changes in pupil sizes after ICL V4c implantation (^*∗*^*P* < 0.05, ^*∗∗*^*P* < 0.01, and ^*∗∗∗*^*P* < 0.001).

Figure 2 shows the pupil size changes in pupillary responses to light. Minimum pupil diameters increased significantly at 1 and 4 weeks postoperatively (both *P* < 0.01) and then returned to baseline at 3 months postoperatively (*P*=0.086). Compared to preoperative values, no differences in maximum pupil diameter were found at 1 week or 1 month postoperatively (all *P* > 0.05); however, maximum pupil diameter significantly decreased at 3 months postoperatively (*P*=0.01). There were significant decreases in initial pupil diameter at 1 week (*P*=0.037), 1 month (*P*=0.001), and 3 months postoperatively (*P* < 0.001), compared to preoperative values.

Preoperative disk halo size was related to SE (*r* = −0.407, *P*=0.008). Disk halo size at 1 week postoperatively was significantly correlated with maximum pupil diameter (*r* = 0.371, *P*=0.016). Disk halo size at 1 month postoperatively was significantly correlated with dilation velocity (*r* = 0.470, *P*=0.002), as well as initial (*r* = 0.325, *P*=0.036), maximum (*r* = 0.365, *P*=0.017), minimum (*r* = 0.307, *P*=0.048), and average pupil diameters (*r* = 0.333, *P*=0.031). Disk halo size at 3 months postoperatively correlated significantly with initial (*r* = 0.446, *P*=0.003), maximum (*r* = 0.483, *P*=0.001), minimum (*r* = 0.425, *P*=0.005), and average pupil diameters (*r* = 0.474, *P*=0.002) (Table 3).

## 4. Discussion

The presence of a halo is a common phenomenon after ophthalmic surgeries. Changes in the presence of a halo must be clearly communicated in discussions of the complications of ICL V4c implantation in clinical practice. In the present study, we examined changes in disk halo size after ICL V4c implantation, as well as factors that influenced halo size.

Disk halo size showed a decreasing trend, but the difference between preoperative and 1-week postoperative values was not statistically significant. This result might be related to postoperative recovery in the early period. Disk halo size decreased significantly at 1 and 3 months after the procedures. Moreover, as in a previous study, preoperative disk halo size was related to the SE [6]. This suggests that the influence of myopia on disk halo size was removed after ICL V4c implantation. Previous studies regarding glare also showed that ICL V4c implantation did not induce a significant additional change in subjective intraocular forward scattering using C-Quant or in the objective scatter index using a double-pass optical quality analysis system at 3 months postoperatively, compared to preoperative values [14, 15]. Notably, Paarlberg et al. observed a significant reduction in the straylight value when using the C-Quant device at 3 months after Artiflex (Ophtec B.V.) phakic IOL implantation [16]. Questionnaire studies also showed that at 3 months postoperatively, the presence of halo was indistinguishable [17, 18].

At 3 months postoperatively, the average disk halo size was 104.76 ± 36.11 arcmin, which is consistent with the reported baseline value of 111.6 ± 39.8 arcmin in healthy subjects [10]. Kamiya et al. reported similar outcomes, in that objective intraocular scattering values in conventional ICL-implanted and ICL V4c-implanted eyes were essentially equivalent to those in healthy eyes [19, 20].

Reductions in postoperative contraction amplitude, contraction velocity, and dilation velocity were observed in this study. The changes in contraction amplitude were consistent with previous studies [8, 9]. In terms of velocity, Totsuka et al. found no significant changes in maximum constriction velocity or maximum dilation velocity, between preoperative measurements and measurements at 1, 3, and 6 months after ICL V4c implantation [21]. This discrepancy with regard to velocity results might be due to differences in measurement precision. These phenomena might represent a potential mechanical impact between the anterior side of the ICL and the posterior iris surface, thus influencing the function of the pupillae sphincter and dilator pupillae. Another explanation might relate to the aqueous humor; a study on aqueous humor dynamics using a computational simulation showed that the flow velocity of the aqueous humor was much greater if the pupil size was smaller after ICL V4c implantation [22]. Thus, aqueous humor resistance might also play a role.

In the present study, the postoperative initial and maximum pupil diameters were significantly smaller than the corresponding preoperative values, and the minimum pupil size returned to the preoperative value at 3 months postoperatively. Totsuka et al. found no significant changes in the pupil diameters of patients aged 31.1 ± 6.8 years, preoperatively and at 1, 3, and 6 months after ICL V4c implantation [21]. Kamiya et al. [23] found that pupil diameters of patients aged 35.7 ± 12.0 years were transiently reduced at 1 day after ICL implantation, then returned to preoperative levels at 1 week after implantation and subsequently remained stable. Two studies in patients who were less than 30 years of age found that the recovery time was more than 3 months [7, 8]. Thus, the occurrence of a reduced pupillary light response might be a short-term change. Additional studies with longer postoperative follow-up periods are needed to further explore changes in pupillary light response after ICL V4c implantation.

Postoperative disk halo size was significantly correlated with pupil sizes from the pupillary response to light. Patients with smaller pupil sizes from the pupillary response to light experienced smaller halos. In a previous study, the disk halo size of myopic eyes demonstrated a relationship with the minimum pupil size from the pupillary response to light [6]. Significant reductions in initial, minimum, and maximum pupil sizes might also help to explain the decreasing trend in disk halo size after ICL V4c implantation.

Furthermore, this study was limited in that it would have been better to record subjective changes in halo phenomena in the form of a questionnaire at each follow-up examination.

In conclusion, after ICL V4c implantation, disk halo size was reduced in the short term. Patients with smaller pupil sizes during pupillary response to light experienced smaller halos after ICL V4c implantation. For patients with large pupils, adequate preoperative communication is particularly important.

## Figures and Tables

**Table 1:** Demographic and refractive data.

Parameters	Mean	SD
Age (years)	24.76	5.16
Sphere (D)	−9.64	2.57
Cylinder (D)	−1.57	1.06
SE (D)	−10.42	2.54
CDVA	1.09	0.10

D = diopters, SE = spherical equivalent, and CDVA = corrected distance visual acuity.

**Table 2:** Time course of halo radius and pupillary responses to light after implantation of ICL V4c.

Variables	Preoperation		Postoperative 1 week		Postoperative 1 month		Postoperative 3 months		*P*
	Mean	SD	Mean	SD	Mean	SD	Mean	SD	
Halo radius (arc minutes)	187.38	86.90	148.33	48.18	122.62	40.55	104.76	36.11	<0.001
Initial pupil diameter (mm)	5.06	0.69	4.96	0.63	4.89	0.63	4.84	0.57	0.005
Amplitude of contraction (mm)	1.87	0.31	1.59	0.30	1.58	0.31	1.58	0.29	<0.001
Latency of contraction (ms)	241.36	66.42	232.26	55.99	230.55	72.94	246.62	54.94	0.603
Duration of contraction (ms)	609.19	84.97	635.86	83.62	654.00	118.25	623.00	94.46	0.302
Velocity of contraction (mm/s)	6.24	0.88	5.07	0.88	4.95	1.03	4.96	0.90	<0.001
Latency of dilation (ms)	850.55	58.26	868.12	71.79	870.64	68.57	867.48	67.23	0.130
Duration of dilation (ms)	1563.57	130.41	1606.64	79.94	1562.29	99.88	1578.67	109.89	0.139
Velocity of dilation (mm/s)	2.51	0.64	2.07	0.25	2.13	0.39	2.11	0.39	<0.001
Maximum pupil diameter (mm)	5.41	0.69	5.32	0.60	5.30	0.56	5.22	0.54	0.021
Minimum pupil diameter (mm)	3.14	0.44	3.31	0.47	3.25	0.42	3.20	0.36	0.001
Average pupil diameter (mm)	4.48	0.62	4.52	0.57	4.49	0.51	4.42	0.46	0.249

*P* represents the *P* values of comparison among preoperative values and postoperative 1-week, 1-month, and 3-month values.

**Table 3:** Correlation analysis between age, spherical equivalent refraction, pupil parameters, and halo radius (arc minutes).

Variables	Preoperation		Postoperative 1 week		Postoperative 1 month		Postoperative 3 months	
	*R*	*P*	*R*	*P*	*R*	*P*	*R*	*P*
Age (years)	−0.072	0.649	0.267	0.088	−0.010	0.950	−0.025	0.875
Preoperative SE (D)	−0.407^*∗∗*^	0.008	−0.088	0.579	−0.211	0.180	−0.233	0.137
Initial pupil diameter (mm)	0.069	0.664	0.230	0.142	0.325^*∗*^	0.036	0.446^*∗∗*^	0.003
Amplitude of contraction (mm)	−0.072	0.649	0.237	0.131	0.285	0.067	0.192	0.223
Latency of contraction (ms)	−0.090	0.571	−0.191	0.225	−0.115	0.467	−0.145	0.361
Duration of contraction (ms)	0.099	0.534	−0.014	0.931	−0.179	0.256	0.037	0.815
Velocity of contraction (mm/s)	0.018	0.912	0.253	0.106	0.252	0.108	0.149	0.345
Latency of dilation (ms)	−0.091	0.567	−0.092	0.564	−0.291	0.062	−0.046	0.773
Duration of dilation (ms)	−0.064	0.686	0.074	0.641	0.278	0.075	−0.088	0.578
Velocity of dilation (mm/s)	0.071	0.653	0.286	0.066	0.470^*∗∗*^	0.002	0.294	0.058
Maximum pupil diameter (mm)	0.169	0.285	0.371^*∗*^	0.016	0.365^*∗*^	0.017	0.483^*∗∗*^	0.001
Minimum pupil diameter (mm)	0.130	0.412	0.164	0.299	0.307^*∗*^	0.048	0.425^*∗∗*^	0.005
Average pupil diameter (mm)	−0.043	0.787	0.221	0.159	0.333^*∗*^	0.031	0.474^*∗∗*^	0.002

SE = spherical equivalent, D = diopters, and *R* = correlation coefficient.

## Data Availability

Data analyzed during the current study are available from the corresponding author on reasonable request.
